# Supplementary material for: Causal Relationship between Aging and Anorexia Nervosa: A White-Matter-Microstructure-Mediated Mendelian Randomization Analysis
Source: Biomedicines. 2024 Aug 16;12(8):1874. doi: 10.3390/biomedicines12081874 (PMC11351342; doi:10.3390/biomedicines12081874)
Supplement: Supplementary file 1 [file biomedicines-12-01874-s001.zip › Supplementary Figure/Figure S4/Singe SNP plot of MR analysis for TL to ubm-b-2042.pdf]

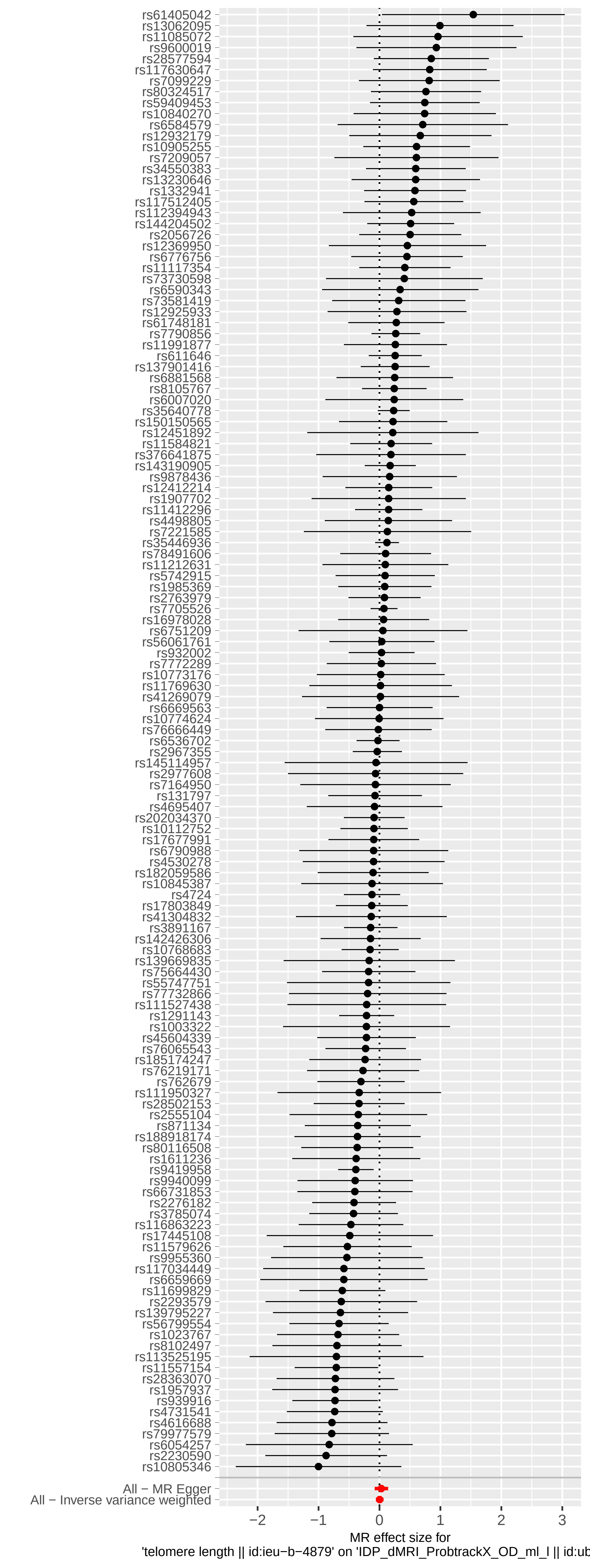
rs61405042
rs13062095
rs11085072
rs9600019
rs28577594
rs117630647
rs7099229
rs80324517
rs59409453
rs10840270
rs6584579
rs12932179
rs10905255
rs7209057
rs34550383
rs13230646
rs1332941
rs117512405
rs112394943
rs144204502
rs2056726
rs12369950
rs6776756
rs11117354
rs73730598
rs6590343
rs73581419
rs12925933
rs61748181
rs7790856
rs11991877
rs611646
rs137901416
rs6881568
rs8105767
rs6007020
rs35640778
rs150150565
rs12451892
rs11584821
rs376641875
rs143190905
rs9878436
rs12412214
rs1907702
rs11412296
rs4498805
rs7221585
rs35446936
rs78491606
rs11212631
rs5742915
rs1985369
rs2763979
rs7705526
rs16978028
rs6751209
rs56061761
rs932002
rs7772289
rs10773176
rs11769630
rs41269079
rs6669563
rs10774624
rs76666449
rs6536702
rs2967355
rs145114957
rs2977608
rs7164950
rs131797
rs4695407
rs202034370
rs10112752
rs17677991
rs6790988
rs4530278
rs182059586
rs10845387
rs4724
rs17803849
rs41304832
rs3891167
rs142426306
rs10768683
rs139669835
rs75664430
rs55747751
rs77732866
rs111527438
rs1291143
rs1003322
rs45604339
rs76065543
rs185174247
rs76219171
rs762679
rs111950327
rs28502153
rs2555104
rs871134
rs188918174
rs80116508
rs1611236
rs9419958
rs9940099
rs66731853
rs2276182
rs3785074
rs116863223
rs17445108
rs11579626
rs9955360
rs117034449
rs6659669
rs11699829
rs2293579
rs139795227
rs56799554
rs1023767
rs8102497
rs113525195
rs11557154
rs28363070
rs1957937
rs939916
rs4731541
rs4616688
rs79977579
rs6054257
rs2230590
rs10805346
All − MR Egger
All − Inverse variance weighted
−2
−1
0
1
2
3
MR effect size for
'telomere length || id:ieu-b-4879' on 'IDP_dMRI_ProbtrackX_OD_ml_l || id:ub
